# Research on the Molecular Interaction Mechanism between Plants and Pathogenic Fungi

**DOI:** 10.3390/ijms23094658

**Published:** 2022-04-22

**Authors:** Lin Li, Xue-Ming Zhu, Yun-Ran Zhang, Ying-Ying Cai, Jing-Yi Wang, Meng-Yu Liu, Jiao-Yu Wang, Jian-Dong Bao, Fu-Cheng Lin

**Affiliations:** 1 State Key Laboratory for Managing Biotic and Chemical Treats to the Quality and Safety of Agro-Products, Institute of Plant Protection and Microbiology, Zhejiang Academy of Agricultural Sciences, Hangzhou 310021, China; 21616143@zju.edu.cn (L.L.); 11816090@zju.edu.cn (X.-M.Z.); wangjiaoyu78@sina.com (J.-Y.W.); baojiandong@gmail.com (J.-D.B.); 2 State Key Laboratory for Managing Biotic and Chemical Treats to the Quality and Safety of Agro-Products, Institute of Biotechnology, Zhejiang University, Hangzhou 310058, China; 12216001@zju.edu.cn (Y.-R.Z.); 11816053@zju.edu.cn (Y.-Y.C.); wangjingyi1996314@163.com (J.-Y.W.); 21816078@zju.edu.cn (M.-Y.L.)

**Keywords:** pathogenic fungi, plants, interaction, penetration, immune response

## Abstract

Plant diseases caused by fungi are one of the major threats to global food security and understanding the interactions between fungi and plants is of great significance for plant disease control. The interaction between pathogenic fungi and plants is a complex process. From the perspective of pathogenic fungi, pathogenic fungi are involved in the regulation of pathogenicity by surface signal recognition proteins, MAPK signaling pathways, transcription factors, and pathogenic factors in the process of infecting plants. From the perspective of plant immunity, the signal pathway of immune response, the signal transduction pathway that induces plant immunity, and the function of plant cytoskeleton are the keys to studying plant resistance. In this review, we summarize the current research progress of fungi–plant interactions from multiple aspects and discuss the prospects and challenges of phytopathogenic fungi and their host interactions.

## 1. Introduction

Pathogenic fungi invade plants in four main steps: adhesion on host surface, form infection structure, invasion of host, colonization and expansion within host. Some pathogenic fungi can even produce metabolites that are toxic to their hosts, and these substances are considered to be one of the main causes of plant diseases. Different pathogenic genes cause different infection processes and metabolic regulation modes. The interactions between plants and pathogenic fungi can be divided into incompatibility and affinity. In non-compatible interactions, local necrotic spots with obvious differences from surrounding healthy tissues are formed at the infection site, namely hyper-sensitive reaction (HR) [1]. In affinity interaction, some fungi take advantage of stomata or trauma on the host surface to invade, usually producing infection structures formed by specialized hyphae. Infection cushion, appressorium, and haustorium help pathogenic fungi invade and establish parasitic relationships with hosts, resulting in plant infection [2].

The innate immune system of plants consists of two main immune responses [3]. One is nonspecific defense response: pattern recognition receptors (PRRs) of plants can recognize highly conserved macromolecular substances common to pathogenic microorganisms which are called pathogen-associated molecular patterns (PAMPs), such as flagella and polysaccharides [4,5]. When PAMPs are recognized by PRRs, relative signal transduction pathways are activated and then induce defense response to limit the invasion of pathogenic microorganisms. This process is called PAMP-triggered immunity (PTI) response [6]. The second is specific defense response: In order to successfully infect plants, pathogenic microorganisms have evolved effector proteins to inhibit the immune response induced by PAMPs. At the same time, plants have evolved R genes to monitor and identify effectors, cause hypersensitive response (HR) and limit the invasion of pathogens. This resistance is called effector-triggered immunity (ETI) [3].

Here, we summarize the current status of the molecular mechanism of the interaction between plants and pathogenic fungi and analyze the molecular mechanism of pathogenic fungi infecting plants and the molecular mechanism of plant immune response in detail from two perspectives.

## 2. Signal Recognition of Pathogenic Fungi Infection Process

Signaling pathway refers to a series of enzymatic reaction pathways that can transmit extracellular signals into the cell through the cell membrane. Receptors on the cell membrane sense external signals. In organisms, these receptor proteins include ion channel receptors, G-protein-coupled receptors (GPCR), tyrosine kinase receptors, and receptors that regulate gene expression. In fungi, heterotrimer G protein participates in the regulation of vegetative growth, pathogenicity, sporulation, and differentiation of infection structure by regulating the activities of adenylate cyclase and phospholipase and ion channels [7,8,9]. In *Ustilago maydis*, heterotrimer G proteins and GPCR are involved in mating and pathogenicity by regulating hormone response and cAMP-dependent signaling pathways [10,11]. In *Aspergillus fumigatus*, the Gα subunit GpaB positively regulates conidia survival and PksP expression in macrophages [12]. Phenotypes of the *gpaB* mutant and the adenylate cyclase mutant *acyA* suggest that a gpaB mediated cAMP-dependent signaling pathway is involved in the pathogenesis of *A. fumigatus* [12,13]. In *Cryphonectria parasitica*, Gα protein Cpg-1 is essential for pathogenesis. Cpg-1 is involved in the growth and sporulation of trophic mycelia, and the pathogenic process is regulated by a pathway independent of Gβγ [14,15,16]. In contrast, Gβ subunit Cpgb-1 positively regulates pathogenicity but does not affect vegetative growth [17]. In *Cryptococcus neoformans*, adenylate cyclase Cac1 positively regulates the formation and toxicity of the capsule [18,19]. On the other hand, Gβ subunit Gpb1 is not required for pod formation, pigment synthesis, or toxicity [20]. Although defective capsular formation is observed in the *gpr4* GPCR mutant, pigment and toxicity remained unchanged [19].

The MAPK cascade pathway is located in the center of the cell signal transmission network, and is involved in regulating cell growth and differentiation, photosynthesis, metabolism, synthesis and release of neurotransmitters, adaptation to adverse environment, infection of pathogens, and other physiological processes [21,22,23,24,25]. In many pathogenic fungi, the HOG pathway mainly plays a role in adapting to high osmotic pressure in the external environment. In *Neurospora crassa*, it is found that the HOG pathway mainly consists of osmotically responsive histidine kinase Os1, histidine phosphate group transfer proteins Hpt1, RRG1/2, and downstream phosphoric acid coupling systems of Os4, Os5, and Os2 [26,27,28]. Studies have found that the HOG pathway in *N. crassa* is not only related to environmental stress, but also regulates the production of bacterial pigments, resistance to diimide fungicides, and pathogenicity of bacteria. The HOG pathway of *Botrytis cinerea* is also reported to be sensitive to osmotic stress, DCFs, and pathogenicity of the pathogen to different hosts [29,30]. However, Osm1 (homologous protein of Hog1), a key element of the HOG pathway in *Magnaporthe oryzae*, is associated with drug sensitivity and osmotic stress, but is not closely associated with pathogen pathogenicity [31,32]. Fus3/Kss1 pathway not only has an important relationship with the sexual reproduction of pathogenic fungi but also plays an important role in regulating the pathogenicity of pathogenic fungi. In *Aspergillus nidulans*, although the scaffold protein Ste5 is missing, it can still form the complex AnSte50-AnSte11-AnSte7-AnFus3. After the complex is activated by upstream signals, AnFus3 will enter the nucleus and activate the activity of AnSteA (homologous protein of Ste12) and AnVeA. Activated AnSteA can regulate mycelial fusion and sexual reproduction of *A. nidulans*, while activated AnVeA can regulate secondary metabolism of *A. nidulans* [33,34,35]. In *M. oryzae*, mutants of *pmk1* (homologous protein of Fus3) cannot produce appressorium and cannot penetrate the host surface [36,37]. The CWI pathway is a kind of MAPK signaling pathway that has been studied well in pathogenic fungi. Kinase Slt2, a core component of the CWI pathway, has been found to regulate cell wall integrity in *Alternaria brassicicola*, *A. nidulans*, and *M. oryzae* [38,39,40,41]. Slt2 has also been found to be significantly associated with the pathogenicity of many pathogens, such as *Candida albicans* [42].

## 3. Regulation of Pathogenic Processes by Transcription Factors of Pathogenic Fungi

Transcription factors are the largest family of trans-acting factors. In a broad sense, all transcription-related proteins except RNA polymerase itself can be classified as transcription factors [43]. The zinc finger protein family is the most widely distributed in the eukaryotic transcription factors family and can generally be divided into zinc finger and zinc cluster structure. Zn2Cys6 transcription factor is a kind of zinc finger protein peculiar to fungi. *Colletotrichum* melanin regulation (CMR) and Pigment of *M. oryzae* (PIG) encodes a protein that contains both zinc fingers and zinc clusters, both of which are involved in melanin synthesis [44]. The AlcR protein of *A. nidulans* contains Zn2Cys6 zinc clusters, and its main function is mainly related to ethanol metabolism [45].

bZIP transcription factors in *A. nidulans* are involved in the regulation of secondary metabolism, sexual reproduction, and stress response [46]. The bZIP protein in *N*. *crassa* is associated with sulfur utilization and oxidative pressure reaction [47,48]. The bZIP transcription factor in *A. fumigatus* mainly regulates asexual reproduction, gelatoxin production, sulfur assimilation, and infection [49,50]. FgAp1, a bZIP transcription factor in *Fusarium graminearum*, is associated with oxidative pressure response and toxin synthesis. FoMeab, a bZIP protein from *Fusarium oxysporum*, is involved in regulating nitrogen cycling pathways [51]. In *M. oryzae*, bZIP transcription factor genes coordinate the physiological processes such as growth and development, conidiation, appressorium formation, infection, and pathogenicity [52].

Homologous heteromorphic box structure transcription factors proteins generally have two protein binding regions, and conformational changes after binding to regulatory proteins; thus, regulating DNA binding activity [53]. In *U*. *maydis*, homologous genes are mainly involved in the regulation of linear growth, sexual reproduction, and infection of host plants [54]. In *Podospora anseria*, the homologous heteromorphic box gene PAH1 regulates mycelia extension growth and male spore production, and its gene deletion mutants grow slowly and the mycelia is compact [55]. In *M. oryzae*, eight homologous heteromorphic box genes are identified and named as MoHox1-MoHox8, in which Δ*Mohox1*, Δ*Mohox4*, and Δ*Mohox6* grew at a slower rate, and aerial hyphae were scarce. Δ*Mohox8* appressorium penetration decreased and pathogenicity decreased. Δ*Mohox7* does not produce functional appressorium in either germ tube or mycelium tip, resulting in a complete loss of pathogenicity [56]. bHLH transcription factors are highly conserved transcription factors in eukaryotes. In *N*. *crassa*, bHLH transcription factor CHC-1 is associated with CO_2_-mediated negative regulation of sporulation [57]. In *A. nidulans*, AnBH1 regulates penicillin synthesis, and DevR regulates sexual and asexual reproduction [58,59]. SclR in *Aspergillus oryzae* promotes sclerotia. EcdR is related to the early differentiation of conidiophore [60,61].

## 4. Virulence Genes Involved in the Infection Process to the Plant in Phytopathogenic Fungi

Different phytopathogenic fungi are evolved to trigger different mechanisms of adhesion to the surface of a host. Without a firm adhesion to the plant surface, penetration by pressure could not proceed successfully even if appressorium is well developed and melanized [62].

### 4.1. cAMP-PKA Pathways

In *M. oryzae*, the PTH11 gene deletion mutants could not form effective appressorium on the hydrophobic surface, and the addition of exogenous cAMP could complement their phenotypic defects [63]. The cAMP signaling pathway catalyzes phosphorylation of target proteins through the activity of cAMP-dependent PKA. Mutation of the CPKA gene delays appressorium differentiation significantly, and in this mutant, only small, non-functional appressorium could be formed, leading to failure in penetration [64,65]. The impairment of *mpg1* mutants in appressorium formation could be remediated by soluble analogs of cyclic adenosyl monophosphate (cAMP) or inhibitors of cAMP-phosphodiesterase [66].

### 4.2. Cell Wall Synthesis and Degradation-Related Genes

Phytopathogenic fungal usually undergo two different strategies when invading host plant tissues: mechanical penetration and enzymatic degradation. Fungal melanin in plant diseases is a general term for a group of biomolecular molecules, which is responsible for the pathogenicity of many phytopathogenic fungi. In classic phytopathogenic fungi *M. oryzae*, three melanin biosynthetic genes *ALB1*, *RSY1,* and *BUF1* have been cloned and mutants of these genes have the phenotype of appressorium turgor loss which results in the loss of pathogenicity [67]. The other key player in mechanical penetration is glycerol. It has been proved that, in appressoria of *M. oryzae*, when in turgor generation, glycerol levels rise drastically [68]. Some other phytopathogenic fungi, such as *Fusarium* or *Cladosporium fulium*, do not undergo morphological changes or differentiate obvious infection structures in the process of host invasion [69]. The appressorium of *Cochliobolus carbonum* has no melanin inside, so it needs to release enzymes to help itself invade the host plant. The SNF1A gene which encodes the cell wall degradation enzyme plays a role during invasion since the deletion mutant strain of the SNF1A gene could not successfully infect the host [70].

### 4.3. Factors Associated with Induction of Plant Defense Tolerance and Degradability

Tolerance and degradation of induced plant defense molecules is one strategy that phytopathogenic fungi usually take. In *M. oryzae*, the ABC1 gene that encodes a protein associated with fungal ATP binding cassette transporters is related to the expansion after invasion, which are thought to be involved in resistance to drugs and phytoalexin [71]. Toxins produced by some phytopathogenic fungi also play an important role in helping colonize the host tissue and they may have adverse effects on a variety of plants. Race T of *Cochliobolus heterostrophus* could produce T-toxin so that it is highly pathogenic on maize while race O, which does not produce T-toxin, is only weakly pathogenic. Tox1 determines toxin production so that it can be regarded as a virulence factor [72].

### 4.4. Autophagy Pathway

It has been shown that autophagy also plays an important role in the infection of pathogenic fungi. Autophagy is an intracellular degradation pathway that is conserved in eukaryotic organisms. *M. oryzae* has been widely used as a model fungus to study the relationship between autophagy and pathogenicity. Deletion of any of the 16 essential genes in nonselective autophagy has a significant effect on pathogenicity (weakened or lost) due to the block in the autophagy process, the reduction in number of conidia, and the disturbance on the maturation of appressoria [73,74,75,76,77,78]. Studies in *F. graminearum* and *Phytophthora sojae* also confirmed the vital role of autophagy-related genes in growth, development, and pathogenicity of phytopathogenic fungi [79,80].

## 5. The Role of Cytoskeletons in the Infection Process of the Plant in Phytopathogenic Fungi

Both microtubules and microfilaments are important parts of the eukaryotic cytoskeleton, which are required in numerous essential cellular processes such as mitosis, endocytosis, mediate fungal vegetative growth, and involve the infection process. Early in 1986, although biochemical mechanism of thigmotropic sensing is not fully understood, the reorganization of both the microtubule and microfilament cytoskeleton in uredospore germlings differentiation has been observed, which is a response to signal reception [81]. Microtubule and F-actin cytoskeleton became reoriented parallel to such scratches on artificial substrates and further demonstrated the evolvement of cytoskeleton in the thigmotropic signal in appressorium formation in *Uromyces appendiculatus* [82]. Besides the participation of cytoskeleton in the early stage of signal perception, when LifeACT-fluorescein fusion protein is used to image F-actin dynamics in developed appressorium, the special F-actin ring formation could be observed in many phytopathogenic fungi such as *M. oryzae* and *C. orbiculare* [83,84]. In the early infection, it is common sense that the classic appressorium pore of pathogenic fungi is a site on appressorium without normal cell wall and melanin to generate penetration peg to realize successful penetration. In *M. oryzae*, the septin ring with four core septins, Sep3, Sep4, Sep5, and Sep6 that is observed at the appressorium pore is demonstrated to be necessary for scaffolding actin, leading to a toroidal F-actin network assembled at the base of the appressorium. Mutation of genes encoding any of the septins is sufficient to lead to failure in infection [85]. F-actin dynamics may vary in different kinds of phytopathogenic fungi depending on different infection mechanisms. For example, F-actin accumulates at the site of the penetration pore in *C. graminicola* while in the center of the appressorium in *A. alternata* [86,87]. In addition, F-actin could form an aster-like structure in appressoria in *Phvtophthora infestans* [88].

## 6. Infection by Pathogenic Fungi Causes Resistance in Plants

Pathogenesis-related proteins (PR proteins), encoded by PR genes, are a wide array of proteins accumulated under the attack of pathogens. Both the hypersensitive response (HR) and systemic acquired resistance (SAR) can activate the PR genes’ expression. Gene expressions of PR-1, PR-2, PR-4, PR-5 families are both upregulated in response to *Fusarium proliferatum* infection in garlic. Another study showed that in rice, OsWRKY67 can directly bind to the promoter of *PR1* and *PR10*, resulting in activation of PR1 and PR10 when facing the blast disease [89,90]. In addition to diseases, abiotic stresses and the related signaling molecules, can also induce the PR gene expression, even the development stage can make a difference [91]. PR genes have different biological functions. The main role of PR genes is to orchestrate response against pathogenic infection. On the other hand, they are also implicated in plant development and differentiation processes, such as germination, cutin synthesis, and somatic embryogenesis [92].

### 6.1. PTI Signal Transduction

Currently, lipopolysaccharide, ergosterol, and glucan in fungi and oomycetes are all plant PAMPs that have been found. In addition to binding PAMPs to stimulate plant PTI response, PRR also acts as a receptor for other external stimuli. For example, two receptors with a LysM domain have been identified in legumes, which are necessary for the symbiosis between plant and Rhizobium [93,94]. BAK1 is a serine/threonine protein kinase and regulates the recognition and transduction of plant immune signals [95].

The MAPK pathway is known to participate in plant PTI reaction mainly through the following pathways. First, MAPK induces the expression of downstream plant immune-related genes. For example, the MEKK1-MKK4/MKK5-MPK3/MPK6 cascade pathway in Arabidopsis mediates plant defense response induced by pathogens by phosphorylating WRKY transcription factors related to disease resistance, including WRKY22, WRKY23, WRKY29, WRKY46, and WRKY53 [4]. Second, MAPK regulates the synthesis of plant antitoxins. When the MAPK cascade pathway was inhibited, the sensitivity of maize to *F. graminearum* was significantly increased while the expression of a large number of genes related to antitoxin synthesis was decreased, and the expression of ZmWRKY79 was also inhibited. Thirdly, MAPK cascade mediates cell wall thickening. The callose accumulation between plant plasmodesmata can strengthen the thickness of cell wall, so as to resist the diffusion and spread of pathogens. Effector avh331 can inhibit the accumulation of callose caused by *Phytophthora* by inhibiting the downstream reaction of the MAPK pathway. In the *ap2cl* mutant, due to the obstruction of the MAPK pathway, the accumulation of callose decreased by 56% compared with the wild type after treatment with elf18. Phosphothreonine lyase can also inhibit callose accumulation triggered by flg22 [96,97]. Fourthly, MAPK can activate plant hypersensitivity, such as overexpression of plant immune-related genes and burst of ROS. It has been proved that when watermelon is infected by *F*. *oxysporum*, it will overexpress CIMKK5 and CIMKK7, and ROS rapidly accumulate to accelerate the death of infected cells, so as to reduce the damage of pathogens to healthy cells. Fifthly, MAPK can promote the stomatal closure of plants and effectively impede the invasion of pathogens. Studies have shown that ABA can activate MAPKs to trigger signal transduction guard cells in pea, and the addition of MAPK inhibitors can significantly inhibit stomatal closure [98]. At the same time, stomatal closure obstruction is reflected in MPK3, MPK6, MKK4, and MKK5 mutants [99], but it seems that MPK3 and MPK6 have functional redundancy in this process [100]. Sixthly, the MAPK cascade is involved in the synthesis of plant disease-resistance-related hormones: phosphatase ap2c1 can inactivate Arabidopsis MPK4 and MPK6 by dephosphorylation and inhibit the synthesis of ethylene, resulting in the decline in plant immune function against *B. cinerea* [101].

**Figure 1:**
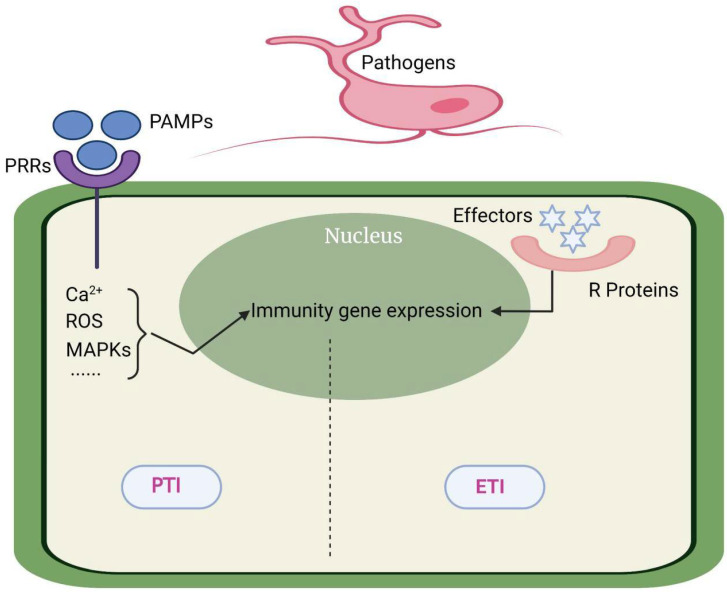
ETI and PTI. PTI is mainly stimulated by PRRs on the surface of pathogenic microorganisms, which can lead to non-specific defense responses (basal defense responses) in plants; plant R proteins recognize effector proteins produced by pathogenic microorganisms and initiate ETI, which can make plants produce specific defense responses.

Ca^2+^ is an important second messenger in the process of plant growth and disease resistance. The largest family of Ca^2+^-sensitive protein kinases in plants is CDPKs, which have a Ca^2+^ binding EF hand motif [102]. It has been found that CDPK4/5/6/11 are involved in the burst of ROS and improving plant resistance to pathogens in Arabidopsis. CDPK5 was proved to accept the stimulation by flg22 and phosphorylate RbohD to promote the burst of ROS [103] (Figure 1).

### 6.2. Molecular Mechanism of Induction of ETI

One of the most important characteristics of the ETI reaction is called gene-to-gene resistance. This theory holds that for each gene that determines plant disease resistance, there is also an Avirulence gene (*Avr*) that determines the pathogenicity of pathogens. When disease resistance genes, *Avr* and R genes, are present together, the ETI response will occur. If one of the three is missing, *Avr* will exert their pathogenicity and destroy the host’s immune system [104,105]. By analyzing the cloned R gene in plants, it is found that the structure of the R protein is relatively conservative, while the structure of *Avr* is changeable. Therefore, the efficient interaction between R protein and *Avr* determines whether the ETI reaction can be normally induced. Up to now, three main modes of interaction between R protein and *Avr* has been demonstrated: direct interaction mode, indirect interaction mode, and transcriptional regulation model [106,107,108].

The interaction between rice and *M. oryzae* is the most typical example of the direct interaction model. Pi-Ta coded by the R gene in rice and the AVR-Pita of *M. oryzae* can interact directly and become the basis of disease resistance. The rice mutant of the LRR domain of pita can destroy the interaction with AVR-Pita, resulting in rice infection [109]. In addition to direct interaction, more and more evidence shows that there are some helper proteins in the host which act as a medium to help the R protein and AVR binding. Up to now, there are three explanations for the mechanism of indirect interaction: guard model, decoy model, and bait and switch model. The guard model has now proved that there are two molecular mechanisms: The first is that the R protein and the target protein are separated under natural conditions. When AVR attacks the target protein, the R protein is activated and binds to the target protein. Second, the R protein is initially bound to the target protein. The attack of AVR protein can separate the R protein from the complex and turn on the related disease resistance pathway downstream. Both models can explain why a small number of R genes can respond to a large number of AVR attacks from a variety of pathogens [107]. If there is no R protein corresponding to AVR in plants, the existence of trap protein can also mislead AVR to bind more to itself, so as to avoid the target protein from being attacked [110]. AvrPto produced by *P. syringae* can recognize and bind FLS2 and EFR1 in Arabidopsis which are important PPRs for plant PTI response, and their attack can destroy the PTI pathway of Arabidopsis. In response to the destruction of AvrPto, Arabidopsis evolved the decoy protein Pto, which competitively binds to AvrPto and activates the hypersensitivity reaction induced by Prf. In other species without Prf, even if ROS burst cannot be successfully induced, the presence of Pto ensures that the sensitivity of plants to AvrPto will not be stronger [108]. The bait and switch model is a supplement to the decoy model, as the decoy model cannot explain that some R proteins have been found to interact directly with AVR in vitro (yeast double hybrid) but indirectly in vivo. This is because the R protein in this model must have the following two characteristics: firstly, the N-terminal can bind to the bait protein, and secondly, its LLR domain can interact with AVR [111] (Figure 1).

### 6.3. Connection and Interaction between PTI and ETI System

For a long time, PTI and ETI have been considered as two independent systems. However, with the deepening of research, PTI and ETI have become interactive from relative independence. There are many intersections and similarities between them in early signal transduction and downstream immune response. Nowadays, Minhang Yuan et al. found that the phosphorylation of NADPH oxidase RbohD promotes the production of ROS, which is an early key signal event connecting PRR- and NLR-mediated immune systems. The phosphorylation of BIK1 in PTI signal transduction is necessary for the complete activation of RbohD, gene expression, and resistance in the ETI system. In addition, NLR signaling rapidly increased the transcription and protein expression of PTI signaling factors [112].

## 7. Plant Cytoskeleton Function during Pathogenic Fungal Infection

The plant cytoskeleton mainly consists of microtubules and microfilaments (actin filaments). Actin filaments are formed by global actin (G-actin) and filamentous actin (F-actin), while microtubules consist of α and β tubulin [113,114]. Both microtubule and actin have been involved in many cellular events, such as mitotic division, molecule and organelle trafficking, and cell wall deposition [115]. In recent years, research has linked the physiological processes of the plant cytoskeleton to the immune system [116].

Actin is reported to control the opening and closing of the stomata [117]. Especially against fungi, the actin cytoskeleton plays an important role in providing physical resistance [118]. In a calm state, the actin cytoskeleton stretches normally to maintain cellular metabolism. After recognizing a pathogen, the actin filament reorganizes and its density increases at the site of infection [119,120]. Actin then acts as a compound to restrict the strength of pathogen and deliver DAMP to surrounding cells to activate the defense reaction [121]. If this polymerization is blocked by treatment with the drug latrunculin B or cytochalasin E, it leads to a higher susceptibility to pathogens. This phenomenon is observed in many species. For example, penetration of *M. oryzae* is promoted by treating with actin agonist cytochalasin E in barley, which suggests that the actin cytoskeleton is involved in providing resistance to pathogens [122].

The microtubule cytoskeleton also changes as pathogens attempt to invade plant cells. Unlike the actin cytoskeleton, the alterations of microtubules have been observed in an early stage of the plant’s response [123]. Early experiments have observed the presence of radial microtubule arrays beneath the appressorium during fungal infection, while the array is disrupted when the pathogen successfully penetrates in [124]. Similarly, pharmacological destruction of the microtubule array results in induced plant susceptibility. Meanwhile, depolymerization of microtubules by using oryzalin increases the expression of defense genes. Interestingly, the microtubules rapidly aggregate when the fine needle presses the leaf and dissipate when they are lifted.

### 7.1. Plant Cytoskeleton Is Involved in Signal Transduction

The cytoskeleton plays an important role in signal transduction [125]. As a most important second messenger in cells, Ca^2^^+^ plays an important role in signal transduction [126]. Large amounts of work have highlighted that the cytoskeleton can act as both upstream and downstream actors of Ca^2^^+^ signaling [127,128]. As an upstream actor, the plant cytoskeleton can adjust Ca^2^^+^ homeostasis. Higher Ca^2^^+^ concentration promotes depolymerization of the cytoskeleton, and the depolymerization of the intracellular skeleton also causes a large influx of extracellular Ca^2^^+^, and cytoskeletal repolymerization prevents the influx of Ca^2^^+^ [129,130]. As the downstream factor, Ca^2^^+^ can directly regulate the stability of the cytoskeleton, or indirectly by regulating calcium-stimulated protein kinases (CDPKs). Moreover, it can also be transmitted by Ca^2^^+^/CaM through microtubule cytoskeleton-associated proteins [131,132]. In Arabidopsis, two PRRs, FLS2 (flagellin receptor) and BRI1 (brassinosteroid receptor) localized on the plasma membrane interact with BIK1 and form complexes to conduct signaling transduction and play a role in immune signaling activation [133]. A recent study showed that the FLS2–BIK1 and BRI1–BIK1 complexes associate with localized microtubule cytoskeleton. The results indicate that the plant cytoskeleton is involved in the formation of functional complexes to activate immune-related downstream signaling [134].

### 7.2. Plant Cytoskeleton Is Involved in Plant Defense Reaction

*ADF4* (actin depolymerizing factor), important for actin turnover, is also required for *RPS5*, which encodes an R protein [135]. Moreover, in *Atadf4* mutant, the activation of two mitogen-activated protein kinases, *MPK3* and *MPK6* that are known to play important roles in the development of PTI and ETI, are inhibited [136]. Meanwhile, increased microfilament density requires the action of BIK1 and BAK1, both of which are important components of PTI [137].

ROS accumulation and hypersensitive response (HR) production are the most important disease resistance mechanisms of plants against fungal infection. Cytoskeleton reorganization may play a crucial role in ROS accumulation and HR production. After treatment with the actin depolymerizing agent cytochalasin A, it was found that the HR production, the production of H_2_O_2_, and the formation of papillae induced by powdery mildew were all significantly inhibited. A similar result is observed when *C. orbiculare* is inoculated after Oryzalin treatment, the accumulation of H_2_O_2_ is weakened. Depolymerization of the actin cytoskeleton also affects the transcription level of pathogenesis-related genes. Treated by cytochalasin E, *PR-1* expression is increased in tobacco. Similarly, the expression of SA-related genes in Arabidopsis also increased after treating with latrunculin B and cytochalasin E [138].

## 8. Summary

Plant disease resistance and pathogenesis are a very complex interaction system. The interaction between plants and pathogenic fungi can be analyzed from two aspects: one is the process of pathogenic fungi infecting plants; the other is the immune response of plants caused by pathogenic fungi after infecting plants. In the process of pathogenic fungi infecting plants, fungi have evolved unique infection structures, including infection cushion, appressorium, and haustorium, so that they can better infect the plant. In the process of infecting plants, pathogenic fungi trigger a series of reactions through surface recognition proteins. One of the most extensively studied is the MAPK pathway. Transcription factors of pathogenic fungi are key factors that cause plant disease. At present, there are many studies on transcription factors, but there are few studies on the role of transcription factors in the interaction system between pathogenic fungi and plants. Therefore, starting from the regulation of transcription factors, it is a research trend to study the interaction between pathogenic fungi and plants. At present, the research on pathogenic factors in pathogenic fungi is relatively in depth, and autophagy is also a key factor affecting the pathogenicity of pathogenic fungi. The cytoskeleton performs many functions in cellular life activities. The cytoskeleton plays a key role in the pathogenic process of pathogenic fungi. Studying the molecular mechanism of these pathogenic factors will provide a theoretical basis for screening and designing new drugs as targets for these proteins in the future. From the perspective of the immune resistance of plants caused by pathogenic fungi infecting plants, the immune responses of plants are mainly divided into ETI and PTI. For a long time, PTI and ETI have had great differences in recognition mechanism and early signal transduction and are considered to be two relatively independent types of systems. However, with the extensive and in-depth research, PTI and ETI have become cross-blooming from relatively independent. The study of how PTI and ETI interact to fight pathogens has also become one of the important scientific issues that need to be solved urgently.

The constitutive expression of disease PR proteins in plants is correlated with plant disease resistance to varying degrees. Therefore, PR proteins in plants play a direct and an important role in plant disease resistance. Plant cytoskeletal actin is widely involved in plant immune responses, and studies have found that chemical damage to actin can increase plant susceptibility. Many pathogens produce effector factors that destroy the integrity of the plant cytoskeleton to achieve an effective strategy for pathogenicity. Such effectors either have the effect of actin depolymerization or can effectively prevent actin multimerization. Can plants recognize the state of actin and fight back? Recent studies have shown that chemical depolymerization of actin filaments can trigger plant resistance to pathogen infection through specific activation of salicylic acid (SA) signaling, dependent on vesicular trafficking and phospholipid metabolism [120]. This suggests that the relationship of cytoskeletal actin in plant–pathogen interactions is more complex than previously thought. Therefore, have plants evolved a mechanism to sense the pathological disruption of actin to trigger a defense response? What is the molecular basis? If not, why does actin depolymerization only specifically affect SA content and not other phytohormones? This will be a new model of actin in plant–microbe interactions and a future research direction.
